# Viruses and Type 1 Diabetes: From Enteroviruses to the Virome

**DOI:** 10.3390/microorganisms9071519

**Published:** 2021-07-16

**Authors:** Sonia R. Isaacs, Dylan B. Foskett, Anna J. Maxwell, Emily J. Ward, Clare L. Faulkner, Jessica Y. X. Luo, William D. Rawlinson, Maria E. Craig, Ki Wook Kim

**Affiliations:** 1 Faculty of Medicine and Health, School of Women’s and Children’s Health, University of New South Wales, Sydney, NSW 2031, Australia; sonia.isaacs@unsw.edu.au (S.R.I.); d.foskett@unsw.edu.au (D.B.F.); a.maxwell@student.unsw.edu.au (A.J.M.); e.ward@student.unsw.edu.au (E.J.W.); c.faulkner@unsw.edu.au (C.L.F.); jessluozmail@gmail.com (J.Y.X.L.); w.rawlinson@unsw.edu.au (W.D.R.); m.craig@unsw.edu.au (M.E.C.); 2 Virology Research Laboratory, Serology and Virology Division, NSW Health Pathology, Prince of Wales Hospital, Sydney, NSW 2031, Australia; 3 Faculty of Medicine and Health, School of Medical Sciences, University of New South Wales, Sydney, NSW 2052, Australia; 4 Faculty of Science, School of Biotechnology and Biomolecular Sciences, University of New South Wales, Sydney, NSW 2052, Australia; 5 Institute of Endocrinology and Diabetes, Children’s Hospital at Westmead, Sydney, NSW 2145, Australia; 6 Faculty of Medicine and Health, Discipline of Child and Adolescent Health, University of Sydney, Sydney, NSW 2006, Australia

**Keywords:** enterovirus, type 1 diabetes, virome, vaccine, antiviral, islet autoimmunity, coxsackievirus, next-generation sequencing, unbiased sequencing

## Abstract

For over a century, viruses have left a long trail of evidence implicating them as frequent suspects in the development of type 1 diabetes. Through vigorous interrogation of viral infections in individuals with islet autoimmunity and type 1 diabetes using serological and molecular virus detection methods, as well as mechanistic studies of virus-infected human pancreatic β-cells, the prime suspects have been narrowed down to predominantly human enteroviruses. Here, we provide a comprehensive overview of evidence supporting the hypothesised role of enteroviruses in the development of islet autoimmunity and type 1 diabetes. We also discuss concerns over the historical focus and investigation bias toward enteroviruses and summarise current unbiased efforts aimed at characterising the complete population of viruses (the “virome”) contributing early in life to the development of islet autoimmunity and type 1 diabetes. Finally, we review the range of vaccine and antiviral drug candidates currently being evaluated in clinical trials for the prevention and potential treatment of type 1 diabetes.

## 1. Type 1 Diabetes

Type 1 diabetes (T1D) is characterised by the chronic immune-mediated destruction of pancreatic β-cells, with affected individuals requiring lifelong exogenous insulin [1,2]. Globally, over 1.1 million children and adolescents under the age of 20 are estimated to have T1D, with approximately 128,900 new cases diagnosed each year. In children 0–14 years, India and USA currently have the highest prevalence of T1D (95.6 and 94.2 thousand cases, respectively) [3]. In Australia, a recent study of T1D incidence in children 0–14 years from 2002 to 2017 found a mean incidence of 25.0 per 100,000, additionally revealing a sinusoidal pattern in incidence over time represented by 5-yearly cycles. Mean incidence also increased with age, with the highest incidence in 10–14-year-olds (224% higher than 0–4-year-olds). Wide geographical variation in the mean incidence of T1D has been described, with incidence increases of up to 6.6% per year in Poland, a levelling off reported in populations such as Finland and Sweden, and a slight decreasing trend in Australia over recent years, particularly in 0–4-year-olds. This variation both between and within countries and different ethnic populations is suggested to reflect geographical differences in genetic susceptibility and environmental risk in addition to disparities in diagnostic criteria including islet autoantibody testing requirements [2,4].

T1D is categorised into four stages: (1) presymptomatic T1D with the presence of multiple islet autoantibodies (type 1a) but normoglycemia; (2) presymptomatic T1D with progression to dysglycaemia; (3) dysglycaemia and clinical symptoms such as polyuria, polydipsia, polyphagia, weight loss, fatigue and diabetic ketoacidosis (DKA)); (4) long-standing T1D [5,6]. Acute and long-term complications of T1D include severe hypoglycaemia, DKA, vascular disease, nephropathy, retinopathy and neuropathy; with lifespan also reduced [7,8,9].

## 2. Islet Autoimmunity

Most T1D is preceded by the development of islet autoimmunity (IA), serologically confirmed by the presence of at least one diabetes-associated islet autoantibody to insulin (IAA), glutamic acid decarboxylase 65 (GADA), protein tyrosine kinase-related islet antigen 2 (IA-2A) and zinc transporter 8 (ZnT8A). IA can appear from around six months of age with incidence peaking prior to two years of age in the genetically at risk but will be generally present months to years before symptomatic onset, reinforcing the need for early-stage interventions and increased monitoring of presymptomatic T1D. These autoantibodies typically appear sequentially rather than simultaneously, making it unclear whether multiple or single events precipitate seroconversion and eventual T1D development [10,11]. The risk of developing T1D increases as additional autoantibodies are detected [12,13,14], with the presence of a single autoantibody (‘early’ IA) conferring a 15% risk of progression to T1D [15], whereas two or more antibodies (‘established’ IA) are associated with an 80% risk of progression to T1D [16,17,18,19,20]. Early seroconversion and increased autoantibody concentrations can be observed in a high proportion of at-risk children, with over 80% of children who developed T1D seroconverting before three years of age [21]. The first-appearing or primary antibody has been proposed to represent two major IA phenotypes representing early or late diagnosis of IA [18,22]. Increasing IAA concentrations have been used to predict progression to overt T1D, with proinsulin highlighted as an important autoantigen in T1D diagnosed in early childhood. Conversely, the appearance of GADA as the primary autoantigen may result in progression at a later age, affecting the design of early interventions [21,23,24].

T1D pathogenesis is marked by selective destruction of insulin-producing cells by effector autoreactive and bystander CD8^+^ T cells, directly contrasted by the action of regulatory T cells. Dendritic cells and even mast cells (although limited data are available) have also been implicated in T1D pathogenesis, as they present islet autoantigens to autoreactive T-cells, resulting in dysregulated peripheral immune tolerance [25]. However, the occurrence of the resulting islet infiltration by autoantibodies (insulitis) is heterogenous amongst islets both within lobules of a single pancreas and between individuals, following a relapsing–remitting nature during early disease and perhaps reflecting the highly variable asymptomatic period in preclinical T1D [26]. Approximately 70–95% of β-cells are usually lost at the onset of symptoms, resulting in a reduced pancreas size, although in some individuals a 40% reduction is adequate to elicit symptoms [2,16,27]. Efforts to preserve any residual β-cell function (measured by C-peptide production) using immune intervention therapies have had limited success [28,29,30,31,32]. Notably, only 15% of children displaying single IA positivity progress to T1D [33], and conversely, only 10% of individuals with T1D display single IA positivity [16]. Therefore, there is an increasing focus on the prevention of T1D progression from the early stages of non-clinical disease. Development of more economical and efficient assays of islet autoantibody detection may allow for more widespread employment of IA screening and potential for use in the general population, enabling earlier diagnosis and intervention [17,34].

## 3. Genetics

Comprehensive genome-wide association studies have identified over 60 genetic loci associated with increased T1D risk, with approximately half of the genetic risk attributed to the human leukocyte antigen (HLA) genotype, with notable contributions also arising from the *INS*, *PTPN22*, *CTLA4* and *IL2RA* genes [35]. HLA-class II DR and DQ allele haplotypes DRB1*03:01-DQA1*05: 01-DQB1*02:01 and DRB1*04-DQA1*03:01-DQB1*03:02 show the highest risk, with DR3/DR4 (DQ2/DQ8) heterozygotes displaying a 30-fold increased risk of IA and T1D in the general population. Whilst a combination of islet autoantibodies has been previously used to predict increased risk of progression to T1D in first-degree relatives [36], we can now use genetic risk scores (GRS) to predict progression to T1D in IA positive children [37]. GRS are calculated using a combination of HLA and non-HLA genes, with weighted values given to both high-risk HLA class II genotypes plus a weighted value assigned to each susceptible allele of HLA class I and non-HLA single nucleotide polymorphisms (SNPs). Individuals with lower GRS experience slower progression to IA, and slower development from both single and multiple IA to T1D in The Environmental Determinants of Diabetes in the Young (TEDDY) cohort [38]. Recent improvements in T1D GRS algorithms have led to the development of T1D GRS2 for standardised use with greater predictive power [39].

Although individuals with a first-degree relative with T1D are at approximately 15-fold increased relative lifetime risk for T1D compared to the general population, over 85% of diagnosed children have no family history, highlighting the major contribution of environmental factors in the aetiology of T1D [5,40]. T1D heritability varies depending on which family member has T1D, with the risk of T1D in the offspring higher with a T1D-affected father (~6%) compared to a T1D-affected mother (~2%). Furthermore, having a dizygotic twin imposes a slightly higher risk of T1D (~10%) compared to a non-twin sibling (~6%), highlighting the role of the intrauterine environment on T1D-risk. [41]. Interestingly, the proportion of individuals with the highest risk genotype DR3-DQ2/DR4-DQ8 has been shown to decrease over time in multiple populations in the United Kingdom, Finland and United States [2].

## 4. Environmental Triggers

An interplay between genetics and environmental factors such as the virome, microbiome and metabolome is suggested to regulate immune tolerance, with the introduction of environmental, lifestyle or dietary exposures currently being investigated as either accelerating or protective [42]. A range of potential environmental triggers has been proposed, including viruses. The hypothesised role of viral infections in the initiation of IA and the progression to T1D is supported by a large body of epidemiological and animal model-based evidence [43,44]. Multiple viruses have been associated with IA/T1D to date, including enterovirus (EV) [45,46,47,48,49,50,51,52,53,54], rotavirus [55,56,57,58,59,60,61], cytomegalovirus [62,63,64,65,66,67,68], Epstein-Barr virus [63,69,70], parechovirus [71,72,73], influenza [74,75,76], parvovirus [77,78], mumps [79,80,81], rubella [80,81,82,83,84,85] and human endogenous retrovirus [86,87,88,89]. By far, the strongest supporting evidence exists for EVs. Our previous meta-analysis of 26 molecular studies and >4400 participants revealed EV infection was 10 times greater at the onset of T1D compared to healthy controls [90]. Furthermore, T1D-specific risk alleles contained within genes involved in immune function have been shown to alter susceptibility to viral infection or affect the extent of the host antiviral response [91]. The rs1990760 SNP within *IFIH1* has been associated with increased detection of EV RNA in blood [92] and separately with severe EV-A71 infection [93]. The rs2476601 SNP within *PTPN22* has been associated with lower IFN production by macrophages in response to TLR ligand stimulation (as would occur during viral infection) [94], and additionally it has been suggested that PTPN22 could suppress the function of effector T cells, diminishing their response to viral infection and allowing the establishment of persistent infection [91,95].

The timing of environmental triggers is likely to be critical, with environmental influences potentially commencing in utero and within the first year of life, emphasising the importance of longitudinal prospective cohort studies that follow at-risk children from pregnancy, such as the Environmental Determinants of Islet Autoimmunity (ENDIA) and Type 1 Diabetes Prediction and Prevention (DIPP)-novum studies [96,97]. Our recent meta-analysis of observational studies revealed maternal viral infections during pregnancy resulted in offspring that were twice more likely to develop T1D (OR 2.16, 95% CI 1.22–3.80; *p* = 0.008), highlighting the need to measure infections in utero as well as during early life. The adoption of large, national or international prospective birth cohort studies allows for the examination of any temporal links between infection in utero and the eventual development of IA or T1D in the offspring [46].

## 5. Enteroviruses

EVs are non-enveloped, single-stranded icosahedral RNA viruses classified within the *Picornaviridae* family that primarily display faecal–oral transmission, within occasional cases of vertical and respiratory transmission also possible [98]. Human EVs are ubiquitous and responsible for serious diseases such as poliomyelitis, myocarditis and aseptic meningitis [99]. However, many EV infections cause subclinical or mild disease and are thus underreported, with a small proportion proceeding to clinical identification [100]. More severe EV infection is typically seen in children and neonates, with proposed intrinsic immunity in the adult mature gut moderating the course of infection and preventing viraemia [101].

There are more than 100 characterised genotypes of human EV, classified into four species: EV-A to -D. Also included within the EV genus are rhinoviruses, which predominately cause upper respiratory tract infections and distinct clinical presentation [102,103,104]. The linear EV RNA genome spans 7.2–8.5 kb in length, consisting of four structural (P1) capsid proteins and seven non-structural (P2 and P3) proteins, forming a single polypeptide which is cleaved by a viral 3C and 2A proteases [105,106]. The EV 5′-untranslated region (UTR) contains internal ribosome entry sites which allow for ribosome recruitment during cap-independent translation of EVs’ single polypeptide. Self-assembly of VP1-VP4 capsid proteins into empty capsid particles and transcription of the positive-strand RNA genome facilitated by non-structural proteins is followed by RNA encapsidation and formation of infectious virions. The mechanism of release is still unconfirmed but is proposed to involve changes to cell membrane integrity, lysis and apoptosis [107]. The 5′ and 3′ UTRs are highly conserved amongst all EV species and have historically formed the basis of primer and probe designs used in molecular diagnostics of general EV infection [108]. The highly variable major capsid protein VP1 codes for genotype-specific determinants of neutralisation and antigenic sites and is therefore typically used for EV genotypic classification [109,110].

EVs invade host cells primarily via the coxsackievirus and adenovirus receptor (CAR), expressed in both α- and β-cells, with entry of genomic RNA post adsorption followed by translation and replication of sense viral RNA in the cytosol in a cap-independent manner [111]. A specific isoform of CAR with a terminal SIV motif and a unique PDZ-binding domain at the C-terminal (CAR-SIV) has been shown to be highly and selectively expressed within β-cells and is localised mainly to insulin secretory granules, which may further contribute to the sensitivity of human β-cells to EV infection [112]. Secretory granule proteins are proposed to be hijacked during exocytosis, allowing internalisation of virus particles by existing endocytic machinery. This is further supported by the identification of viral replication complexes around insulin granule membranes in coxsackievirus B (CVB)-infected human islets using electron microscopy [113,114].

## 6. Historical Association between EV and T1D

The viral aetiology of T1D was first proposed in the mid-1920s, describing a seasonal variation of T1D onset with peaks in diagnoses occurring in the colder months, reflecting that of seasonal viral variation [115,116] and was then revisited decades later in relation to CVB infection in 1969 [117]. The seasonality of T1D diagnosis has been since confirmed in larger studies in nonequatorial regions [118,119,120]. The Finnish DIPP cohort revealed the appearance of islet autoantibodies in a seasonal pattern, with IA following the seasonality of viral infection [44]. A combination of factors in addition to viral infection, including higher inflammation, altered diet, reduced exercise and reduced vitamin D levels, has been suggested to influence the seasonality of T1D and other autoimmune diseases [121,122].

Whilst viral infection alone may not be sufficient to induce T1D in any individual, a number of factors such as timing, site, viral load, strain and type of infection, in combination with host genetics and the pancreatic microenvironment has been highlighted as critical for disease pathogenesis [123]. There are at least 26 EV genotypes historically associated with IA and T1D, with members of EV-B species such as CVB and ECHO genogroups most frequently described [102,124]. In children with high-risk T1D genotypes from the TEDDY study, coxsackievirus A (CVA) genotypes were the most frequently detected EVs in stools, with genotyped EVs representing EV-A, EV-B and at a lesser scale, EV-C species (61.5%, 38% and 0.5%, respectively). CVA4, enteric cytopathic human orphan virus (ECHO) 18 and ECHO26 genotypes demonstrated the longest period of viral shedding. Interestingly, children who were HLA-DQ2/8 heterozygous were slightly less frequently EV positive, compared to children homozygous for HLA-DQ2 or HLA-DQ8. Compared to the general population, genotypes within the EV-B species are more often reported in children with symptomatic and severe infections [125].

A higher viral titre and multiplicity of infection have been proposed to determine T1D induction rather than any defined diabetogenic phenotype [126]. A prediabetic state at the time of EV infection increases the risk of developing T1D, as shown by studies of mice with pre-existing insulitis. Inoculation of older non-obese diabetic mice with high doses of CVB3 variants triggered sudden diabetic onset from both highly pathogenic and poorly pathogenic strains, suggesting that many EV strains have the potential to induce T1D in predisposed individuals if encountered at a sufficient dose [127]. Perhaps the most compelling evidence supporting the role of EV in T1D pathogenesis was provided by the live biopsies of pancreata from six adults with recent T1D onset, which found evidence of EV infection in all individuals [128] and enhanced islet antiviral immune responses [129,130,131]. These findings are further supported by recent data from the network for Pancreatic Organ Donors (nPOD) Virus Group [132,133,134].

A temporal association between EV infection and the appearance of islet autoantibodies was reported in the DIPP cohort, with CVB1 also associated with increased risk of IA and T1D whereas CVB3 and CVB6 were associated with reduced IA risk. Whilst EV infection within six months of IA development was implicated using viral RNA detection in blood [135], a more recent study of EV RNA in stool revealed children with IA had more infections compared to control children, with most of these infections occurring at least 12 months prior to the development of the first autoantibody. EV genotypes most commonly reported in stools of case children were CVA2, 4 and 16 [136].

## 7. Pathogenesis Mechanisms

**Figure 1:**
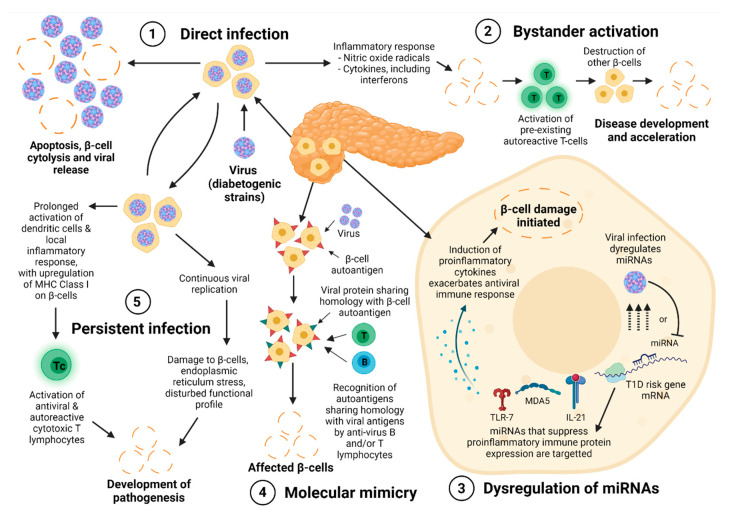
Putative mechanisms for initiation of islet autoimmunity (IA) and acceleration to type 1 diabetes (T1D) by viruses: (**1**) Direct infection and β-cell cytolysis; (**2**) Bystander activation; (**3**) Dysregulation of host miRNAs; (**4**) Molecular mimicry; and (**5**) Persistent infection. These mechanisms are not mutually exclusive and likely act in interacting pathways to trigger IA and/or facilitate progression of T1D pathogenesis [107,137]. However, there is little direct mechanistic evidence from humans, with poor understanding of how virus-induced insulitis may involve into a targeted autoimmune attack [102]. Abbreviations: MHC, major histocompatibility complex.

EVs have been proposed to induce T1D through a number of non-mutually exclusive mechanisms: direct cytolysis, molecular mimicry, bystander activation, persistent infection and microRNA dysregulation (Figure 1). The type of infection is thought to be a key determinant of T1D pathogenesis. Direct cytolysis of β-cells resulting from a lytic infection can trigger an inflammatory reaction that further promotes IA by releasing either pre-existing autoimmune effector T cells (bystander activation) or β-cell autoantigens [137,138,139]. Molecular mimicry can occur when viral peptides exhibit high homology to islet peptides, such as similar epitopes expressed between 2C protease of EV and human islet autoantigen GAD-65, with cross reactivity leading to the presentation of viral antigens which activate antiviral or autoreactive T-lymphocytes [48,102,137]. Following entry into the host cell, EVs are recognised by pattern recognition receptors including toll-like receptors and melanoma-differentiation associated protein 5 (MDA5) [140]. This process activates downstream JAK/STAT, NF-kβ and MAPK pathways, resulting in the release of pro-inflammatory cytokines and chemokines [141,142]. This can then induce apoptosis through the activation of downstream caspases and the release of autoantigens in a highly inflammable state. This state produces an upregulated major histocompatibility complex (MHC) class I expression, further promoting autoimmunity [105,143]. Epitope spreading allows recognition of further autoantigenic epitopes as a result of β-cell damage and associated stress, in a cyclic process stimulating autoreactive T cells [144]. EV infection is additionally associated with a humoral immune response prior to and at diagnosis of T1D [145]. An additional mechanism contributing to EV dissemination in persistently infected β-cells has also been recently suggested, with a non-lytic method of egress using β-cell-derived extracellular vesicles. Interestingly, extracellular vesicle-mediated EV infection was not inhibited by virus-specific neutralising antibodies, highlighting its application in immune evasion [146].

Viral persistence is an alternative putative mechanism underlying the EV-mediated pathogenesis of T1D. Persistent EV infections can lead to the prolonged activation of the immune system, resulting in a continuous presentation of viral peptides and production of proinflammatory cytokines that progressively promotes IA development. Through a prolonged state of inflammation including increased release of type I interferons (IFN) [133,147,148], resulting in endoplasmic reticulum stress in β-cells, persistent viral infections have the capacity to evoke antiviral and autoimmune responses [107,134,149,150]. This is compounded by defective viral clearance by natural killer (NK) cells, with cytolytic activity of NK cells also demonstrated towards persistently infected β-cells [151,152]. The distinction between lytic and persistent EV strains has previously been shown to be strain-specific rather than serotype-specific, suggesting the role of viral genetic factors. Selection of a less or non-cytopathic strain and a reduced replication rate are frequently reported factors [153,154]. CVB3 and 4 establish intestinal persistence, particularly in mucosal lymphocytes, as evidenced by long-term detection of viral RNA and VP1 in infected mice, with these lymphocytes indicated as the principal reservoir for viral spread to other organs such as the heart and pancreas. CVB3 replication in B lymphocytes was additionally responsible for early viral excretion in stools and the chronic release of infectious viral particles throughout the duration of infection [155]. Persistent EV infection of intestinal mucosa was demonstrated in individuals with T1D undergoing mucosal biopsies for gastrointestinal complaints in Finland, with EV RNA detected using in situ hybridisation more frequently in cases compared to controls (74% vs. 29% respectively, *p* < 0.001). Viral RNA was frequently detected in the absence of viral protein, suggestive of defective viral replication. EV RNA was detectable in cases even after 12 months, demonstrating that a large proportion of individuals with T1D display a persistent EV infection associated with an inflammatory response within gut mucosa [153].

Viral adaptation processes such as the establishment of persistence, modulation of viral replication and immune evasion may be facilitated by viral genomic modifications obtained during intra-host evolution. This is highlighted by the divergence of EV genotypes as a direct result of high mutagenic rates of ~4–8 mutations/10^4^ nucleotides during viral replication from the lack of proofreading activity in their RNA-dependent RNA polymerase [156,157,158,159]). Terminal deletions within the 5′ UTR facilitated the establishment of viral persistence in the pancreas of mice by diminishing viral replication and translation, and terminally deleted EV RNA was detected in human heart tissue [160,161,162,163,164]. Viral forms lacking certain genomic RNA secondary structures can result in the loss of viral ribonucleoprotein complexes, which are important in regulating viral genomic replication. Disruption of secondary structures in this domain has been further associated with impaired viral RNA sensing by retinoic acid inducible gene I (RIG-I) and MDA5 receptors, supporting a mechanism of immune evasion [164].

In vitro studies using parallel infectious clones of a lytic E9-DM strain of ECHO9 isolated from a T1D-affected child demonstrated that an amino acid substitution at T81A in the VP1 region led to a decrease of virulence, creating a benign rather than destructive infection [165]. Indeed, mutations in the VP1 capsid protein of multiple EV genotypes such as EV-A71 have been associated with increased virulence, changes in receptor specificity and cellular tropism [166,167,168,169,170]. A multitude of SNPs were recently identified in persistently passaged clinical and prototype strains of CVB1 in pancreatic ductal and beta cell lines (PANC-1 and 1.1B4), spanning both structural and non-structural genes. Most interestingly, one mutation affecting the VP1 capsid protein canyon region (K257R) was found in all persisting strains and is predicted to influence EV interaction with decay-accelerating factor (DAF) during internalisation. Other accumulating mutations were identified at BC, DE and EF loops and the C-terminus of VP1, the puff region of VP2, the knob region of VP3 and an infection-enhancing epitope of VP1. Furthermore, long-term passage of CVB1 resulted in the production of smaller viral plaques, highlighting the potential for persistence to reduce the cytopathic effect of EV infection due to changes in translation, adsorption or internalisation. This reinforces the importance of the capsid region during viral persistence, with the potential to identify hallmarks of persistency as an ultimate goal [159].

Another factor that may contribute to viral pathogenesis of T1D is the role of microRNAs [171,172,173]. We previously demonstrated that CVB5 infection leads to the significant dysregulation of multiple microRNAs that regulate the expression of a network of T1D risk genes in human pancreatic islets. This adds yet another layer of complexity in the mechanisms underlying EV-mediated T1D pathogenesis [174].

## 8. Site of Infection: Gut, Pancreas and Respiratory

The body route and site of infection are also important determinants as to whether EVs elicit the initiation of IA and/or accelerate progression to T1D [175]. Persistent EV infection of intestinal and blood cells may potentiate these additional sites as chronic reservoirs from which secondary infection of the pancreas or other organs such as the heart may occur [107]. The anatomic intersection of the lower gut and pancreatic lymphatic drainage in the pancreatic lymph nodes poses another mechanism for the activation of β-cell autoimmunity. EV replication initially occurs in the intestinal mucosa before the virus disseminates into the lymphatic system, circulating to other organs including the pancreas [176,177]. CVBs may infect intestinal epithelial cells by evading the host immune response, blocking the production of type I and III IFNs [178]. Eventual pancreatic infection is supported by the detection of EV RNA in pancreatic tissue obtained from individuals with both recently diagnosed and long-standing T1D [111]. Specific immunostaining of pancreatic islets detected EV protein in 61% of individuals with recent-onset of T1D versus 6% of controls [45], demonstrating the tropism of EV for human islets which is highlighted by the strong expression of CAR in pancreatic islets but not exocrine pancreas [179]. In the DiViD study, EV protein and hyperexpression of HLA-I molecules were detected in the islets of all six participants, with EV RNA detected in four of the six cases. Furthermore, the small proportion of VP1-positive islets (1.7%) and low titre of EV RNA further support the notion of a low-grade viral persistence [128]. Interestingly, viral infection is not limited to endocrine tissue, with viral infection of exocrine tissue such as acinar cells resulting in innate immune activation and inflammation, priming nearby β-cells for destruction [26].

Multiple EV species infect the respiratory tract; EV-C species as well as EV-D68 are found only in the respiratory tract, and rhinoviruses are responsible for over 50% of all upper respiratory tract infections (RTIs) worldwide [180,181]. A growing body of epidemiological data supports the role of RTIs in the development of IA and T1D [22,182,183,184,185,186]. This includes a recent report from the All Babies in Southeast Sweden (ABIS) cohort, demonstrating that maternal RTIs during pregnancy, particularly in the third gestational month, significantly increase the risk of T1D in the offspring (OR 4.1, 95% CI 2.2–7.5; *p* < 0.001) [187]. Current data on the association of RTIs and T1D are limited to self-reported or clinically diagnosed history of infections, with molecular data for RTIs in IA/T1D cohort studies lacking [22,47,183,187,188,189,190]. The proposition of the respiratory tract as an alternate source of primary infection resulting in secondary pancreatic infection is not out of the question, with higher rates of RTI recently linked to increased risk of IA in at-risk children. Parent-reported respiratory infectious episodes (RIE) in the TEDDY cohort revealed an association between higher rates of RIE in a 9-month period and higher risk of IA (p < 0.001), highlighting the importance of surveillance during early life and the time window preceding seroconversion to IA. Types of IA-associated infections included the common cold, influenza-like illness, sinusitis and laryngitis/tracheitis, with RIEs reported in winter resulting in 42.4% increased IA risk. This suggests that children with frequent RTI are at highest risk of progression, although misclassification of infection via measurement using RIEs remains as a major limitation and prevents the detection of subclinical infection obtained using molecular-based techniques [22]. Future demonstration of EV infection in multiple body sites from the same individual with IA or T1D (e.g., in the gut, respiratory tract and blood virome), particularly in longitudinal samples, would provide further support for the association between EV and IA/T1D.

## 9. Leaky Gut

Alterations in gut permeability as a direct result of viral infection, combined with the presentation of novel antigens to intestinal draining lymph nodes, can eventually lead to the appearance of autoreactive CD8^+^ T-cells primed with cross-reactive epitopes in the pancreatic lymph nodes [26]. Changes to gut permeability involving decreased expression of integral membrane proteins Claudin-1 and occludin were demonstrated in diabetes prone rats, with microbial structures such as bacterial lipopolysaccharide and viral nucleic acid proposed to affect epithelial cell function by binding to Toll-like receptors on epithelial cells, causing increased gut permeability. Children with IA or T1D exhibit increased gut permeability (also known as a ‘leaky gut’) and enhanced intestinal inflammation [153,191,192,193,194,195,196]. Moreover, the disruption of the gut epithelial barrier by enteric viral infections may activate β-cell-specific autoimmunity in pancreatic lymph nodes, highlighting the role of the gut as a regulator of insulitis. T-cells activated in the gastrointestinal tract home into islets via mucosal homing receptor MadCAM-1, with luminal antigens processed by pancreatic lymph nodes, suggesting that microbe-derived antigens may trigger local immune cells in the pancreas via bystander activation. Antiviral cytokines and viral proteins also affect barrier function, highlighting the potential role of chronic or recurrent EV infections in the acceleration of immune-mediated destruction initiated in an inflamed gut [197,198].

## 10. Evolution of Virus Detection

Virus detection methods for research and diagnostics have undergone multiple transformations in the past century, particularly revolutionised by the advent of next-generation sequencing (NGS) in the past decade. Traditionally, earlier studies relied on cell culture-based methods to demonstrate the presence of infectious EV in tissues, serum, cerebral spinal fluid and alimentary-tract samples including throat swabs, rectal swabs and stool samples. However, there is no single cell line that can support the growth of every EV, with specialised media and conditions required for different EV types, making culture-based methods difficult, laborious and slow [109,199,200]. Alleviating many limitations of culture-based methods, serological diagnosis of EV infection became widely popular. This could be achieved through neutralisation assays, complement fixation and enzyme-linked immunosorbent assays, which enabled the detection of multiple EV species at once. These methods, however, remain significantly less sensitive, labour-intensive and slower compared to nucleic acid detection methods such as qPCR [110].

Although qPCR remains as the gold standard for rapid and sensitive detection of specific virus targets, there is a limit to how many viruses can be targeted simultaneously through multiplexed primers, and it relies on the preservation of the primer binding sites within the viral genome, which may be disrupted through mutations or recombination events. Such limitations have driven the demand for more comprehensive, high-throughput and untargeted methods for detection and characterisation of complex viral populations [109]. In the current NGS era, near-complete EV genome sequence can be characterised rapidly through amplicon-based sequencing, enabling in-depth examination of intra- and inter-host sequence variations that may contribute importantly to the diabetogenicity of specific EVs [159,201]. Furthermore, recent advances in target enrichment methods have drastically helped to overcome previous bottlenecks of conventional virome sequencing, where an overwhelming abundance of non-viral nucleic acid from the human host, bacteria, fungi and bacteriophage significantly reduced the sensitivity for human viruses [202,203]. Currently, comprehensive, unbiased and sensitive detection of all viruses known to infect humans and all other vertebrates (the “virome”) can be achieved without prior culture through virome capture sequencing (VirCapSeq-VERT). This particular target enrichment NGS method uses approximately 2 million long oligonucleotide probes that hybridise to genomic sequences of all known vertebrate-infecting viruses to enhance the viral sequences themselves in proportion to bacterial and host reads. Sensitivity of VirCapSeq-VERT is on par with or greater than that of qPCR, increasing the recovery of viral reads by up to 10,000-fold compared to conventional metagenomic sequencing [204,205]. The ability to detect all viruses simultaneously without an a priori hypothesis prevents the introduction of any potential investigation bias toward specific viruses, such as the bias toward EVs evident in most previous studies.

Another NGS-based method that allows comprehensive profiling of past infections against all human viruses is VirScan [206]. By measuring the presence of antibodies generated against all past and current viral exposures (humoral response) using a bacteriophage display library of linear viral peptides covering the entire proteome of 1,276 viral strains from 206 human virus species, VirScan enables confirmation of previous viral infections independent of viral nucleic acid or protein. In other words, this method may be useful to detect viral infections that have been already cleared from the body or if viral load is too low at the time of sampling. To date, this cutting-edge tool has proven effective in elucidating novel mechanisms of measles virus infections [207], establishing that maternal transfer of antiviral antibodies occurs very early in gestation [208], and determining the viral aetiology of acute flaccid myelitis [209] and hepatocellular carcinoma [210]. VirScan has yet to be utilised in T1D research.

## 11. Infant Virome

To date, most virome studies have focused on the adult population; the adult gut virome exhibits higher individual and temporal stability, with ~80% of viruses persisting for 1–3 years [211,212]. A better understanding of the infant virome is essential for elucidating the potential role of viruses in the development of disease in children, as well as their influence on bacterial populations and the wider microbiome [213]. The infant virome is dynamic and varies highly between individuals and over time. Despite this, common interpersonal trends exist with respect to virome development during infancy, including eukaryotic virus expansion and the contraction of bacteriophage populations [214]. Furthermore, different locations within the body such as the gastrointestinal tract, oral tract and respiratory tract each provide their own unique microenvironment, with vast differences in virome composition due to microenvironmental differences [212,214,215,216].

It is debated whether the fetus develops in a sterile environment, with recent studies suggesting frequent placental colonisation by bacteria; however, no studies have investigated this theory with regard to the virome [217]. A stepwise assembly of the infant virome has been suggested, with healthy neonates usually born lacking a gut virome; studies analysing neonatal meconium have found that viruses are undetectable in most meconium samples [216,218]. Colonisation of the gut is initiated by bacteria containing integrated prophages, resulting in the eventual production of prophages in early months. The early infant virome is directly influenced by breastfeeding [219], affecting phage distribution, with human-infecting viruses not typically detected prior to until 3–4 months of age. Mode of birth (vaginal vs. caesarean section) is also important, with infants born via vaginal delivery showing greater viral diversity; however these findings have been contradicted in a separate study [220,221,222]. Mother-to-infant transmission of the virome has been proposed from breast milk, supported by significant homology between bacteriophage sequences detected in breast milk and stool viromes [219,223,224]. Additional factors may influence the composition of the gut virome in infancy, such as gender [225], geographical setting [226], the presence of older siblings [225], contact with furry pets [227,228] and antibiotic use as similarly reported for the bacterial microbiome [227,228,229,230].

Whilst a low abundance of eukaryotic viruses is expected in early infancy, eukaryotic viruses such as anelloviruses and parvoviruses are more frequently detected in the gastrointestinal tract as maternal immunity is progressively depleted [214,215,231]. Non-enveloped viruses are most frequently detected in human faeces due to their ability to survive the acidic stomach environment and dehydrating nature of the large intestine. Phages such as crAssphages (cross-assembly phage, member of *Caudovirales*) and also members of *Microviridae* have been identified as the most abundant type of virus in the mature gut [220,232,233]. Given the limited early persistence of vertebrate-infecting viruses, it could be suggested any persistent infections or colonisations that occur may be important. A growing body of evidence suggests that an altered virome composition, especially in infancy, influences long-term health and alters the risk of chronic conditions such as T1D [42,211,212,234,235,236,237]. Despite the probable involvement of virome dysbiosis in IA and T1D development, the exact genotypes of diabetogenic viruses and the importance of timing and duration of virus exposure remain poorly understood.

## 12. Virome and T1D

The first T1D virome investigation used NGS to characterise the virome in plasma samples collected from TEDDY children with rapid-onset T1D (Table 1) [238]. This was followed by an investigation of the longitudinal gut virome changes preceding IA in DIPP children who progressed to T1D [203]. Somewhat contradictory to prior associations identified between EV-B and IA/T1D through use of targeted molecular virus detection methods [90], both studies found no significant associations between the virome and T1D. However, both studies concluded poor sensitivity of conventional NGS compared to targeted qPCR as a key limitation of their findings and a potential source of discrepant results. Soon after, another group investigated the gut virome preceding seroconversion to IA in at-risk children of the DIABIMMUNE cohort [239]. This study also applied conventional NGS but incorporated prior isolation of viral nucleic acid from virus-like particles (VLPs). Yet again, no correlation between EVs and IA/T1D was observed. However, *Circoviridae*-related sequences were inversely associated, suggestive of their potential protective effects. Interestingly, significant differences in the bacteriophage population (beyond the scope of this review) were observed between cases and controls, with higher Shannon diversity and richness observed in controls. However, such differences were not evident in an earlier examination of bacteriophages in DIPP children with a greater sample size [240]. The recent gut virome data from TEDDY represent the largest longitudinal infant virome dataset to date, even outside of the T1D field [150]. In contrast to previous gut virome studies, which by comparison to TEDDY included far fewer longitudinal timepoints and smaller sample size, a statistically significant association between EV-B and IA was found, and a stronger association between consecutive shedding of EV-B and an increased risk of IA (OR 3.70, 95% CI 1.90–7.22, *p* = 0.0001). However, in addition to conventional NGS, the investigators deliberately cultivated viruses isolated from stool suspensions on a mixture of virus-susceptible cells (Hela, Vero, HEK-293 and RD expressing CAR) to boost sensitivity for EVs by amplifying the virus signal. Therefore, although all viruses sequenced in both primary and cultured virome data were considered a combined dataset, inclusion of the culture-NGS approach introduced inherent bias towards EVs and other culturable viruses.

The most critical limitation of conventional NGS for virome applications is its poor sensitivity for eukaryotic viruses, due to signals drowned out by noise from an overwhelming background of non-viral or bacteriophage nucleic acid. In an attempt to boost the sensitivity specifically for vertebrate-infecting viruses, we and others have applied VirCapSeq-VERT to characterise the gut and blood virome of children with IA/T1D [188,205]. Through target enrichment, we observed greater than 3-fold higher overall positivity of viruses in children compared to the DIPP virome study and achieved high concordance with targeted qPCR results. Even with increased sensitivity, no significant association was observed between EV positivity and IA. However, looking beyond indifferences in virus frequency, which are potentially masked by low sample numbers, a large number of viruses were identified as differentially abundant between the gut of cases and controls, including five EV-A genotypes. This indicated a previously unrecognised association of IA with higher EV-A abundance in the gut of children with a first-degree relative with T1D [205]. VirCapSeq-VERT has since been successfully used to elucidate distinct pregnancy gut virome profiles in women with T1D versus without [241] and between infants born from mothers with T1D versus without in the ENDIA study in the first year after birth [242].

Our recent systematic review and meta-analysis of the above observational studies (published by June 2020) that applied NGS to investigate potential associations between early life virome and IA/T1D reported a small, but significant association between IA and consecutive EV positivity in longitudinal samples (OR 1.55, 95% CI 1.09–2.20, *p* = 0.01) [54]. This was largely influenced by the recent TEDDY data. Since then, one additional T1D virome study has been published, reporting on the gut virome profiles of 73 children and adolescents from four geographically distant non-European countries shortly after T1D onset [243].

Overall, inconsistencies in results across existing T1D virome studies (Table 1) could be largely attributed to substantial differences in sampling methods and intervals, sample size including number of participants and/or samples, sample preparation, sequencing approach, different choice of contig assemblers [244,245] and the ever-evolving updates to bioinformatic tools and reference genome databases used for taxonomic classification of viral sequence reads. It is also plausible that some viruses trigger IA/T1D as a hit-and-run event, making it difficult to detect traces of infection [246]. Furthermore, a lack of prospective sampling during pregnancy (maternal) and immediate days after birth in pre-existing T1D cohorts has so far precluded the assessment of potential in utero viral exposures. This gap will be filled by current and future virome investigations in ENDIA and DIPP-novum studies, respectfully.

**Table 1:** Summary of NGS studies to date investigating the virome in association with IA and/or T1D, comparison of study design and virus enrichment methods.

Study; Cohort (Location); Recruitment *	Case/Control Numbers, Inclusion Criteria, Matching Strategy and Sample Numbers (Case/Control)	Sample Type/Collection; Virus Enrichment Strategy; Virus Detection Threshold	Main Findings
Lee, 2013 [238]; TEDDY (USA, Finland, Germany Sweden); High-risk HLA	14 Persistent ≥ 1 Ab+ children with rapid-onset T1D (within 6 months of seroconversion) 14 Controls matched for age, clinical centre, T1D family history 56 samples total (28/28)	Plasma (before and at seroconversion) Conventional NGS only Threshold not stated	No significant associations between the virome and T1D Viruses not detected more frequently in cases versus controls Similar infectious histories in cases and controls Concluded poor sensitivity of conventional NGS compared to targeted qPCR
Kramná, 2015 [203]; DIPP (Finland); High-risk HLA	19 Persistent ≥ 2 Ab+ children who seroconverted at <2 years 19 Controls matched for date/place of birth, sex, HLA 96 samples total (48/48)	Stool (3, 6 and 9 months before seroconversion) Conventional NGS with physical enrichment Virus-specific PCR for confirmation 50p100K raw reads minimum	Virome composition showed no association with IA Most frequently detected human viruses included EVs and parechoviruses Concluded poor sensitivity of conventional NGS compared to targeted qPCR
Cinek, 2017 [240]; DIPP (Finland); High-risk HLA	18 Persistent ≥ 2 Ab+ children who seroconverted at <2 years and progressed to T1D 18 Controls matched for date/place of birth, sex, HLA 92 samples total (46/46)	Stool (3, 6 and 9 months before seroconversion) Conventional NGS with physical enrichment 50p100K raw reads minimum	No significant differences in bacteriophage population in cases versus controls Most frequently occurring bacteriophage (CrAssphage) correlated with Bacteroides dorei, but not other members of Bacteroides Concluded poor sensitivity of conventional NGS compared to targeted qPCR
Zhao, 2017 [239]; DIABIMMUNE (Finland, Estonia) High-risk HLA	11 ≥ 1 Ab+ children 11 controls matched for age, country, sex, HLA, mode of delivery 220 samples total (114/106)	Stool (longitudinal, monthly from 0–3 years) Isolation of VLPs, followed by conventional NGS Threshold not stated	No correlation between eukaryotic viruses and IA/T1D was reported Children are exposed to a broad range of eukaryotic viruses; EV, kobuvirus, parechovirus, parvovirus, and rotavirus sequences most often detected Higher proportion of bacteriophage sequences in controls (*p* = 0.017) Higher abundance of Circoviridae sequences in controls; suggestsprotection from T1D (*p* = 0.026) Virome of cases less diverse than controls as a group, highlighted by differences in the bacteriophage population (*p* < 0.0001)
Hippich, 2018 [188]; BABYDIET (Germany); High-risk HLA	20 ≥ 1 Ab+ children with past respiratory infection 20 controls matched for age 102 samples total (51/51)	PBMCs (3-monthly from 3 months old) Virome-enriched NGS using VirCapSeq-VERT Threshold not stated	No significant associations between the virome and IA Viruses only identified in 1 of 102 samples, which was a rotavirus sequence in a child case Highlighted the challenges of identifying viruses in blood
Kim, 2019 [205]; VIGR (Australia); ≥1 FDR with T1D	Stool 20 persistent ≥ 1 Ab+ children 20 controls matched for age, sex 64 samples total (32/32)	Stool and plasma (before and/or at seroconversion) Virome-enriched NGS using VirCapSeq-VERT Virus-specific qPCR for confirmation 2 thresholds: (1) 100 viral reads matched at species level; (2) 50p100K raw reads	No significant differences in virus positivity or frequency of specific viruses in cases versus controls anellovirus, EV and picobirnavirus were the most often detected viruses 129 viruses differentially abundant in gut of cases and controls, including EV-A genotypes CVA2/5/6/8/14, and EV-B genotypes ECHO30, CVB3 more abundant in cases; suggests viral load may influence IA risk Sensitive analysis with higher virus positivity compared to other studies
	Plasma 41 persistent ≥ 1 Ab+ children 41 controls matched for age, sex 118 samples total (59/59)		
Vehik, 2019 [150]; TEDDY (USA, Finland, Germany Sweden); High-risk HLA	IA case/control 383 persistent ≥ 1 Ab+ children 383 controls matched for age, clinical centre, sex, T1D family history 8564 samples total (4237/4237)	Stool (monthly from 3–48 months, quarterly thereafter) Cell culture-based EV enrichment Conventional NGS with physical enrichment on mixture of cultured and non-cultured VirMAP aggregate bit-score of 400 set as threshold	Proportions of viruses as 72% bacteriophages, 20% verterbrate-infecting viruses and ~8% diet-related viruses (mostly plant viruses) Significant association between EV-B and IA was found Association between consecutive shedding of EV-B and an increased risk of IA (OR 3.70, 95% CI 1.90–7.22, *p* = 0.0001). Number of consecutive stools positive for EV-B (i.e., persistence) associated with IA (OR 3.05) but not T1D Independent, short-duration infection not associated with IA/T1D Human mastadenovirus-F associated with IA (OR 1.33) Early-life Human mastadenovirus-C infections associated with IA protection (OR 0.49)
	T1D nested case/control 112 cases diagnosed with T1D 112 controls matched for age, clinical centre, sex, T1D family history 3380 samples total (1690/1690)		
Cinek, 2021 [243]; (Azerbaijan, Jordan, Nigeria, Sudan); New onset T1D	73 cases with recently diagnosed T1D 105 controls matched for age, place of residence 177 samples total (73/104)	Single stool sample shortly after T1D onset Isolation of VLPs followed by conventional NGS Virus-specific RT-PCR for confirmation Virus positivity determined as 0.001% of total read counts, or 1p100K	No clear and consistent association with T1D was observed Picornaviruses most often observed No significant differences in frequency of eukaryotic viral genera or species in cases versus controls Total read count of eukaryotic viral genera was higher in cases versus controls (OR 1.24) More frequent endogenous retrovirus signal detected in cases versus controls when the threshold was positivity lowered to any mapped read (OR 4.55)

* High-risk HLA genotypes include DR3/4, DR4/4, DR3/3; Abbreviations: 1p100K, 1 viral read per 100,000 raw reads; 50p100K, 50 viral reads per 100,000 raw reads; Ab+, autoantibody positive; DIPP, Type 1 Diabetes Prediction and Prevention; DR3, DRB1*0301-DQA1*0501-DQB1*0201; DR4, DRB1*0401/02/04/05/08-DQA1*0301-DQB1*0302/04; DR3/4, heterozygous genotype comprising both DR3 and DR4 haplotypes; EV, enterovirus; FDR, first-degree relative; HLA, human leukocyte antigen; IA, islet autoimmunity; PBMCs, peripheral blood mononuclear cells; qPCR, quantitative polymerase chain reaction; RT-PCR, real time polymerase chain reaction; T1D, type 1 diabetes; TEDDY, The Environmental Determinants of Diabetes in the Young; VIGR, Australian Viruses in the Genetically at Risk; VirCapSeq-VERT, Virome Capture Sequencing Platform for Vertebrate Viruses.

## 13. Antiviral Vaccines and Therapeutics for T1D Prevention

**Figure 2:**
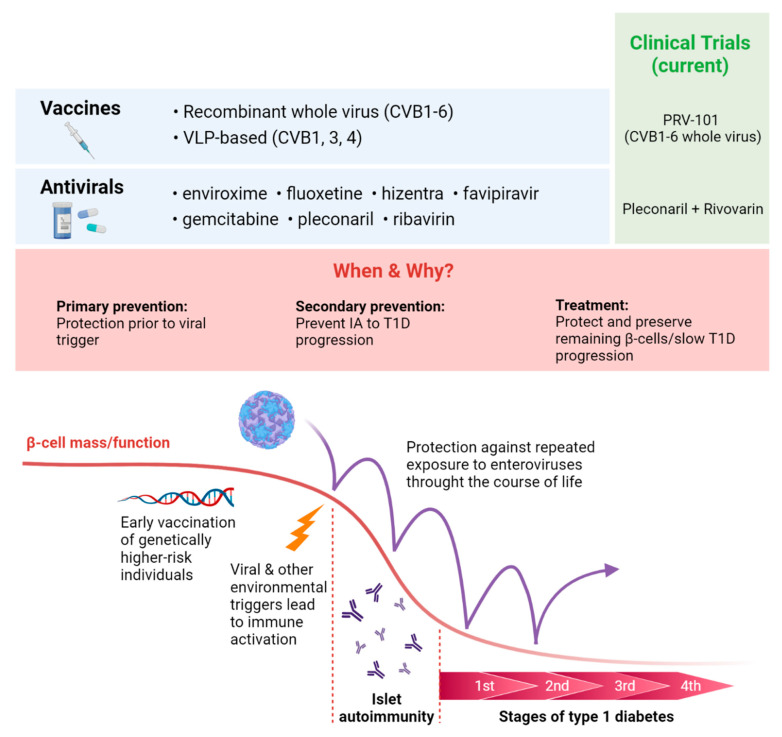
Summary of current antiviral drugs and vaccine candidates for type 1 diabetes (T1D) prevention and potential treatment, illustrating at what stages of islet autoimmunity (IA) and T1D development these may prove useful, indicating current clinical trials aimed at preventing enterovirus infection for prevention or treatment of T1D. Abbreviations: VLP, virus-like particles; CVB, coxsackievirus B.

Driven by the strong rationale for developing antiviral therapeutics to prevent T1D [247,248], there are multiple EV vaccines and antivirals currently being pursued against putative diabetogenic CVB types, some undergoing clinical trials (Figure 2). Most recently, the company “Provention Bio, Inc.” (NJ, USA) launched a first phase randomised clinical trial of the PRV-101 vaccine (January 2021; NCT04690426), the PROtocol for Coxsackievirus VaccinE in Healthy VoluNTteers (PROVENT) trial, to evaluate its immunogenicity and safety in healthy adults. PRV-101 is a hexavalent vaccine developed using formalin-inactivated whole CVB viruses [248,249], specifically designed to prevent CVB1-6 infection, and thereby potentially delay or prevent the development of IA/T1D. Although the use of whole virus vaccines (live and inactivated) has proven their safety and efficacy against poliovirus [250], it is expensive, slow to develop, lacks flexibility to engineer new epitopes and is limited by the culturability of the target EV. 

To overcome such limitations, an alternative vaccine strategy has been explored for CVB1, 3 and 4, involving the use of empty VLPs as antigens [251,252,253,254]. Generated from recombinantly expressed viral structural proteins, VLPs resemble the native CVB capsid structure but lack the infectious RNA genome. Thus, VLP-based vaccines can be manufactured without culturing the virus, are modified rapidly with ease and are not subject to safety concerns associated with live virus vaccines. However, it remains to be determined whether VLP-based vaccines can offer a sufficient level of immunogenicity and protection against CVBs compared to whole virus vaccines in humans. Regardless of strategy, all vaccines predominantly rely on the host to develop sufficient neutralising antibodies against the targeted virus. Therefore, they should ideally be administered to at-risk individuals prior to any exposure to diabetogenic viruses. For individuals already exposed to EVs and potentially harbouring a persistent EV infection, the use of antiviral drugs may offer secondary prevention.

Broadly speaking, antivirals can be divided into two categories: (i) those directly targeting viral proteins (reviewed for EVs in [255]); and (ii) others targeting human host proteins which are integral for viral infection, replication and release [114]. Currently, no antiviral drugs are licensed for the treatment of EV infection. To date, the closest drug to be approved by the U.S. Food and Drug Administration for anti-EV use is pleconaril, which targets the EV capsid. It was considered a common cold treatment but was denied approval due to safety concerns [256]. Recently, a systematic screen of ten clinically used antiviral drugs for their efficacy against CVBs identified hizentra, enviroxime, pleconaril, ribavirin and favipiravir as promising repurposing candidates for T1D intervention trials. These antivirals proved effective against multiple CVBs in their therapeutic serum concentrations in vitro [257]. Subsequent investigation from the same group demonstrated in vitro eradication of persistent CVB1 infection in human pancreatic ductal cells by enviroxime, fluoxetine, hizentra and pleconaril [257]. This is consistent with the previous report that fluoxetine eradicates persistent CVB4 infection in the same cell line, halting replication through inhibition of the viral protease 2C [258]. Additionally, gemcitabine, which binds to the viral RNA-dependent RNA polymerase 3Dpol, has been shown to be an effective inhibitor of broad-spectrum EVs and in combination with ribavirin, exhibits a synergistic antiviral effect on CVB3 and EV-A71 [259].

Antiviral drugs could offer an option for preventing/treating T1D by eradicating infections by diabetogenic EVs. Currently, individuals newly diagnosed with T1D are being recruited into the DiViD and Intervention Trial in Norway (EU Clinical Trials Register EudraCT No. 2015-003350-41). This represents the first randomised clinical trial with antiviral drugs to test the hypothesis that a six-month-long treatment with pleconaril–ribavirin combination can eliminate persistent EV infection in the pancreas.

## 14. Conclusions

Altogether, a plethora of molecular and epidemiological evidence supports a strong rationale for the development of antiviral vaccines for T1D prevention. However, unanswered questions remain regarding which genotypes to target and whether other important viruses have been missed due to the substantial investigation bias towards EVs in previous studies using targeted virus detection methods. To address such concerns, a growing number of studies are applying unbiased NGS approaches to characterise the virome in diverse at-risk populations. One key area that is unknown is whether maternal virus exposure during pregnancy significantly influences the risk of virus exposure and IA development in the offspring, which may be elucidated by studies such as ENDIA and DIPP-novum.

## Data Availability

Not applicable.
